# Ultrastructural study confirms the formation of single and heterotypic syncytial cells in bronchoalveolar fluids of COVID-19 patients

**DOI:** 10.1186/s12985-023-02062-7

**Published:** 2023-05-19

**Authors:** Shikha Chaudhary, Ravi P. Yadav, Shailendra Kumar, Subhash Chandra Yadav

**Affiliations:** 1 Electron Microscope Facility, Department of Anatomy, All India Institute of Medical Sciences, New Delhi, Delhi 110029 India; 2 Department of Anaesthesiology, Pain Medicine and Critical Care, All India Institute of Medical Sciences, New Delhi, Delhi 110029 India

**Keywords:** Acute respiratory distress syndrome, Scanning and transmission electron, Type 2 pneumocyte, Neutrophil, SARS-CoV-2, Bronchoalveolar lavage fluids, Syncytia

## Abstract

**Background:**

SARS-CoV-2 was reported to induce cell fusions to form multinuclear syncytia that might facilitate viral replication, dissemination, immune evasion, and inflammatory responses. In this study, we have reported the types of cells involved in syncytia formation at different stages of COVID-19 disease through electron microscopy.

**Methods:**

Bronchoalveolar fluids from the mild (n = 8, SpO2 > 95%, no hypoxia, within 2–8 days of infection), moderate (n = 8, SpO2 90% to ≤ 93% on room air, respiratory rate ≥ 24/min, breathlessness, within 9–16 days of infection), and severe (n = 8, SpO2 < 90%, respiratory rate > 30/min, external oxygen support, after 17th days of infection) COVID-19 patients were examined by PAP (cell type identification), immunofluorescence (for the level of viral infection), scanning (SEM), and transmission (TEM) electron microscopy to identify the syncytia.

**Results:**

Immunofluorescence studies (S protein-specific antibodies) from each syncytium indicate a very high infection level. We could not find any syncytial cells in mildly infected patients. However, identical (neutrophils or type 2 pneumocytes) and heterotypic (neutrophils-monocytes) plasma membrane initial fusion (indicating initiation of fusion) was observed under TEM in moderately infected patients. Fully matured large-size (20–100 μm) syncytial cells were found in severe acute respiratory distress syndrome (ARDS-like) patients of neutrophils, monocytes, and macrophage origin under SEM.

**Conclusions:**

This ultrastructural study on the syncytial cells from COVID-19 patients sheds light on the disease’s stages and types of cells involved in the syncytia formations. Syncytia formation was first induced in type II pneumocytes by homotypic fusion and later with haematopoetic cells (monocyte and neutrophils) by heterotypic fusion in the moderate stage (9–16 days) of the disease. Matured syncytia were reported in the late phase of the disease and formed large giant cells of 20 to 100 μm.

**Supplementary Information:**

The online version contains supplementary material available at 10.1186/s12985-023-02062-7.

## Introduction

Syncytia are unusually large multinucleated cells generated by the fusion of two or more similar/different types of cells under external/internal influencers such as molecules/virus infection [1]. The plasma membrane of two or more cells fuses to form a single lipid bilayer, resulting in the cytoplasm’s merger but not the nucleus [2, 3]. Naturally, the syncytia formation was mediated by fusogen proteins and was reported in the muscle fibers, placental barriers, and bone osteoclast [3–5]. However, mechanistically, it was induced by virus-mediated cell fusion, where the membrane of enveloped viruses fuses with the plasma membranes of cells. Several common cold coronaviruses, such as hCoV-HKU1, hCoV-NL63, and hCoV-229E were reported to form syncytia in cell culture models, but there is no report for *in vivo* syncytia formation [6, 7].

Syncytia formation is also observed in cell cultures and the tissues infected with SARS-CoV-1, MERS-CoV, or SARS-CoV-2 [8–15]. SARS-CoV-2-induced syncytia were reported in the autopsy samples of lung tissue in severely infected COVID-19 patients [1, 12, 14, 16–20]. SARS-CoV-2 induced the formation of syncytia *in vitro* [21–24] as well as *in vivo* [12, 16, 20, 22], with the fusion of pneumocytes and lymphocytes in the lungs [12, 20].

The generation and type of cells involved in the fusion of syncytial cells at the different stages of the COVID-19 disease were sparingly reported. This report addresses these gaps to strengthen the understanding of syncytial cell formation in COVID-19 patients by employing microscopy-based approaches at the ultrastructure level using PAP (Papanicolaou), IF (Immunofluorescence), SEM (Scanning Electron Microscopy), and TEM (Transmission Electron Microscopy) imaging. We have reported the presence of single and multiple-origin syncytial cells in the BALF (bronchoalveolar lavage fluids) of COVID-19-induced ARDS-like (acute respiratory distress syndrome) patients.

## Materials and methods

### Material

Karnovsky’s fixative (0.5% glutaraldehyde + 2.0% paraformaldehyde), hematoxylin, eosin, orange G., Scott’s water, xylene, DPX, PBS, poly-L-lysin, epoxy embedding kit, and DAPI were purchased from Sigma chemical company, MO, USA. BSA, ethanol was from Himedia, and Triton X-100 was from Fisher Scientific. Osmium tetroxide was procured from Ted Pella, USA, Uranyl acetate from TAAB, UK, and lead citrate from Ladd. Polyclonal anti-SARS-CoV-2 specific primary antibody (Cat no. ab275759) and Alexa fluor-594 conjugated anti-rabbit secondary antibody (Cat no. ab150080) were purchased from Abcam, Plc, UK.

## Methods

### Study design and sample collection

BALF samples were collected from intubated SARS-CoV-2 positive patients in the COVID-19 wards, AIIMS, New Delhi. All samples were collected between 3rd October 2020 and 31st January 2021 (Additional file 1: Supplementary Table 1). The patients were categorized into three groups based on the Indian Council of Medical Research guideline (ICMR) [25]. These group were (A) mild infection non-ARDS patients (non-ARDS, n = 8; SpO2 > 95%, no oxygen support, only upper respiratory tract symptoms with mild fever and without shortness of breath or hypoxia, 2–8 days after first symptoms), (B) moderate infection (n = 8, respiratory rate ≥ 24/min, breathlessness, and SpO2 level 90% to ≤ 93% on room air, external oxygen support, 9–16 days after first symptoms), and (C) Severe ARDS-like patients (ARDS-like, n = 8; respiratory rate > 30/min, breathlessness, SpO2 level < 90% on room air, and admitted to HDU/ICU with external oxygen support, 17th day onwards)[26, 27]. There was very high variability in the patient’s response to the infection and level of COVID-19 disease with respect to the days after the infection. The maximum number of patients were recorded with a mild disease-like symptoms within 2–8 days, moderate disease-like symptoms within 8–16 days, and severe disease-like symptoms > 17 days after infection [26, 27]. Thus, we collected the samples from only those patients who fulfilled disease conditions (ICMR guideline) and timelines criteria for this study [25]. RT-PCR test was performed to confirm the COVID-19 infection for all the patients recruited in the study (recorded but not included).

### Sample processing for the cellular enrichment

The BALF sample (15-20ml) was primarily fixed in freshly prepared 20 ml, 2X Karnovsky’s solution (final 1% glutaraldehyde + 4.0% formaldehyde) in 0.2 M phosphate buffer and stored at 4°C in a COVID-19-designated refrigerator. An on-duty medical physician reviewed and cross-checked all patient’s medical records. Patients’ demography, symptoms and signs, RT-PCR results, radiographic findings, treatment, supportive care, and survival data were recorded but not included (Additional file 1: Supplementary Table 1).

After the primary fixation, the BALF solution was diluted ten times with 0.1 M NaCl solution and strained through a nylon mesh cell strainer with a 100 μm pore. The filtrate was centrifuged at 2500 rpm for 3 min in a swinging bucket, and excess mucus was removed by washing cell pellets (2–3 times for 10 min) with PBS solution. The cellular content was enriched by centrifugation at 1200 g for 5 min and resuspended again in the primary fixative A (0.5% glutaraldehyde 2.0% paraformaldehyde in 0.1 M PB buffer). These samples were processed for PAP staining, IF, SEM, and TEM.

### PAP staining

For PAP staining, cytosmears were prepared using 200 µl of BALF at 800 rpm for 5 min on poly L-lysine coated glass slides and preserved in 90% ethanol. The smears were washed in water for 1 min, and hematoxylin staining was done for 2–4 min with one-minute washing under running tap water, Scott’s water, and again tap water, respectively. The smears were gradually dehydrated in 70%, 95%, and 100% ethanol for 2 min each, treated with Orange G for 4 min, and progressively dehydrated. The smears were dipped in Eosin stain for 10 min and treated with 95% ethanol and acetone for 2 min each. Finally, the stained smears were treated with xylene (3 times, 5 min each) and mounted in DPX.

### Immunofluorescence using SARS-CoV-2 spike protein-specific antibody

For the Immunofluorescence study, BALF samples were washed with 0.1 M phosphate buffer (3 times), and smears were prepared with 10 µl of sample on poly-L-lysine coated glass slides and air-dried at RT. Smears were permeabilized by PBST (0.1% Triton X-100 in 1X phosphate buffer saline, PBS) and incubated with a blocking solution (2% BSA in PBS) for 30 min. After blocking, it was incubated with the primary antibody (Abcam ab275759, polyclonal antibody against S1 spike protein, dilution 1:500) for four hours in a humid chamber at room temperature. After washing with PBS, fluorophore-conjugated secondary antibody (Alexa fluor-594 conjugated anti-rabbit secondary antibody, Abcam, Cat No.- 150,080; dilution 1:500;) was added and incubated for an hour at RT in the darkroom. Smears were washed with phosphate buffer, and DAPI (1 µg/mL) was added and incubated for 5 min. Excess DAPI was removed by washing with PBS, and smears were mounted with 90% glycerol. Fluorescence imaging was performed on a laser scanning confocal microscope (Leica SP8, Germany).

### Scanning electron microscopy

For SEM, enriched and primary fixed cellular components of BALF were dehydrated with ethanol and critical point dried (E-3100, Quorum Tech). Samples were mounted on double-sided tape on the aluminum stubs and were sputter-coated with a gold-based sputter coater (HHV BT-150) for 180 s. Electron micrographs were obtained on EVO18 (Zeiss, Germany) SEM operated at 20 kV accelerating voltage, 8–10 mm average working distance with a secondary electron (SE) detector, and magnifications ranging from 5,000X to 30,000X.

### Transmission electron microscopy

For TEM imaging, enriched cellular constituents of BALF were primarily fixed with 2.5% glutaraldehyde + 2.0% paraformaldehyde in 0.1 M phosphate buffer (PB). The cellular pellets were washed with 0.1 M PB (pH 7.4) and post-fixed with 1% osmium tetroxide in 0.1 M PB (pH 7.4) for one hour (secondary fixation) at 4°C. Pellets were washed with distilled water for 2 h and stained with en bloc staining with 2% uranyl acetate in 50% ethanol. These samples were again washed with distilled water and dehydrated with an ethanol series (50%, 70%, 80%, 90%, and 100%). Dehydrated pellets were infiltrated with toluene/resin and embedded in Araldite CY212 resin. The blocks were polymerized at 65°C for 48 h. Ultrathin sections (~ 70 nm) were prepared using UC7 ultramicrotome (Leica) and mounted on grids. Sections were stained with 5% uranyl acetate and 5% lead citrate and imaged using Talos F200 Transmission Electron Microscope (Thermo Fisher Scientific).

## Results

Cell-enriched BALF samples of mild, moderate, and severe ARDS-like patients (n = 8 each) were fixed with Karnovsky’s solution and imaged for PAP (cytosmear), IF (S protein-specific antibody for infection level), SEM (surface ultrastructure), and TEM imaging (ultrastructural information). We could not find any syncytia in the mildly infected patients (Additional file 1. Figure S1). However, single and different cellular origin syncytial cells with distinct morphology and shape were observed in moderate and severe ARDS-like patients. We have found that mostly type II pneumocytes, neutrophils, monocytes, and macrophages fused to form the syncytial cells in the BALF. The shape varies from rounded to oval and sizes from 20 to 100 μm.

### Microscopic confirmation of syncytial cells from type-II pneumocytes

**Fig. 1:**
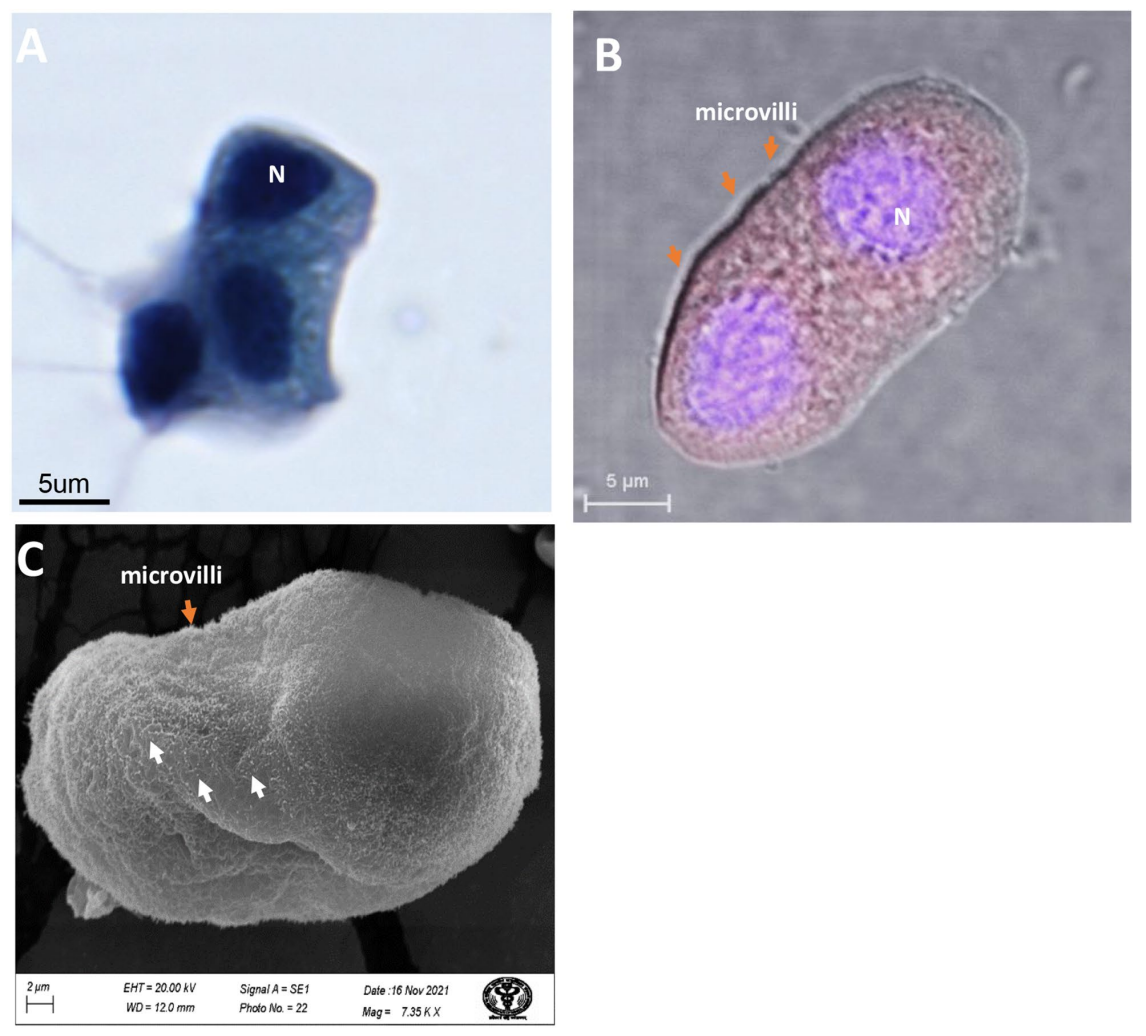
Syncytial cells generated from the homotypic fusion of type II pneumocytes of COVID-19 patients in the moderate stage of the disease (between 9–16 days after the first symptoms). **(A)** The multinucleated dense nucleated and vacuolar cells cytoplasm confirms the type II pneumocytes under PAP imaging. **(B)** The complete membrane fusion of type II pneumocytes (two) with moderate immunofluorescence using SARS-CoV-2 spike protein-specific primary antibody indicates the presence of the virus. **(C)** The SEM image (surface ultrastructure) of type II pneumocytes showed small microvilli-like structure, characteristics of type-II pneumocytes. N, nucleus; white arrow, SARS-CoV-2 virus; orange arrow, microvilli.

We have identified multinucleated syncytial cells generated by type II pneumocytes in moderately infected patients. PAP imaging showed reactive cellular changes with nuclear enlargement and cytoplasmic condensation in type II pneumocyte-originated syncytia. The multinucleated, dense, and vacuolar cells cytoplasm confirms the syncytial cells with type II pneumocytes origin. These cells showed moderate immunofluorescence with SARS-CoV-2 spike protein-specific antibody, indicating the presence of the virus. SEM imaging revealed a surface morphology with small microvilli, a characteristic feature of pneumocyte type II cells (Fig. 1). We could not find such syncytia under TEM that might have revealed a better understanding of the ultrastructural changes after syncytia formation.

### Syncytial cells of neutrophils origin showed a higher level of infection

**Fig. 2:**
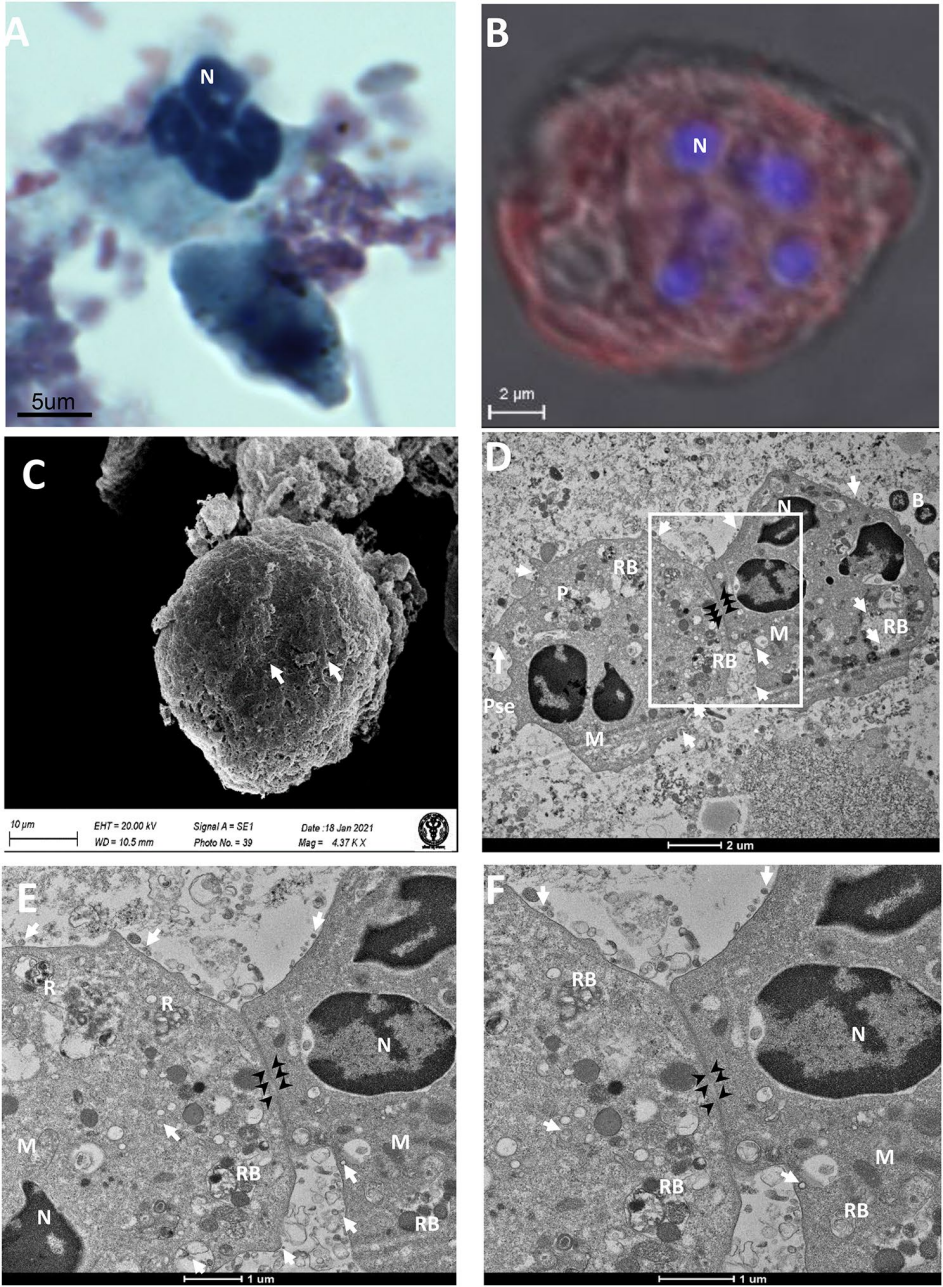
Syncytial cells of neutrophils origin showed the plasma membrane fusion initiation. (**A)** PAP imaging of syncytia with the dense nucleus and granular cytoplasm confirming the neutrophil origin. (**B)** Neutrophil origin syncytial cell with multiple nuclei and immunofluorescence confirming SARS-CoV-2 infection **C**. SEM image of neutrophil origin syncytial cell of size 40 μm. The rough surface with plasma membrane protrusions with plenty of virus-like particles (white arrow) confirmed the neutrophil origin. (**D, E & F)** low and high magnification TEM images showing initiation of syncytia formation (membrane fusion). N, nucleus; M, mitochondria; P, phagosomes; RB, residual body; B, bacteria; Pse, pseudopodia; white arrow, SARS-CoV-2 virus; black arrowhead, the site of the fusion of membrane.

We have also observed the syncytial cells of neutrophil origin under PAP, IF, SEM, and TEM in a moderate disease condition. PAP imaging identified the syncytial formation of two neutrophils with multiple nuclei (not multilobed nuclei) (Fig. 2A). Immunofluorescence studies confirm the severe cellular infection and presence of the SARS-CoV-2 virus. The multinucleated cells with typical neutrophil signatures confirm their origin (Fig. 2B). SEM imaging revealed a syncytial cell of ~ 40 μm with the characteristic feature (rough surface with membrane protrusion) of neutrophil origin (Fig. 2C). TEM imagining showed the initiation of plasma membrane fusion with two adjacent neutrophils (Fig. 2D). These membrane fusions initiation points were clearly visible at a higher magnification, which may be an essential initial step for syncytial cell formation after virus infection (Fig. 2E-F). Multiple immature and mature viral particles were observed in the cytoplasm of these cells (arrows).

### Heterotypic syncytial cells were also formed at moderate stages of COVID-19 disease

**Fig. 3:**
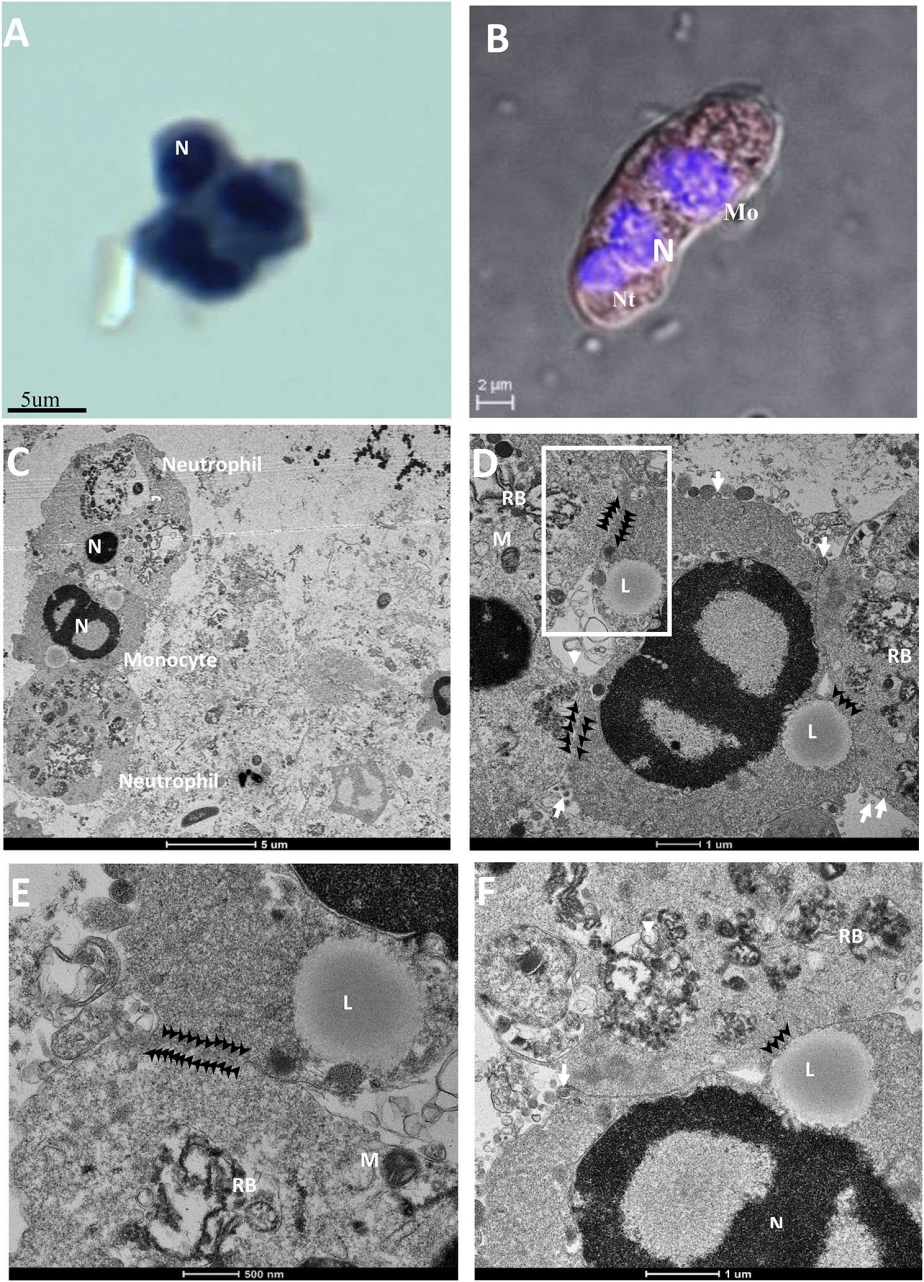
Syncytial cells formed by multiple origins of cells of COVID-19-induced ARDS patients. (**A**) PAP imaging of monocyte and neutrophil origin with multilobed and horseshoe shape nucleus. (**B**) Multilobed nuclei and centric nuclein confirm the neutrophil and monocyte cells, respectively, with a moderate level of SARS-CoV-2 infection in IF. (**C-F**) Membrane fusion between monocytes and two adjacent neutrophils under TEM imaging with different magnifications. Monocyte was identified due to the presence of active lipid bodies and a central nucleus. The neutrophilic granules and phagocytes confirm the neutrophil origin of the adjacent cells. N, nucleus; M, mitochondria; RB, residual body; L, lipid body; white arrow, SARS-CoV-2 virus; black arrowhead, the fusion site of the plasma membrane.

At the moderate stages of the disease, several heterotypic syncytia from neutrophil-monocytes fusion were observed. These cells were observed in PAP, IF, and TEM imaging. IF imaging confirms syncytial cell formation with neutrophils and monocytes with moderate to severe SARS-CoV-2 infection (Fig. 3B-C). TEM revealed the initiation of plasma membrane fusion with adjacent cells (black arrowheads) at multiple places. The monocytic origin cells (middle cells) showed characteristic ultrastructural features such as a bilobed nucleus, the presence of a lipid body, and phagocytic granules (Fig. 3D-G). The ultrastructure features include numerous primary azurophilic (large and electron-dense), secondary specific granules (small, light staining), pseudopodia in the plasma membrane, highly folded cell membrane, and a large number of phagocytes of the adjacent cells to monocytes confirming them as neutrophilic granulocytes. These cells showed a relatively higher number of matured viruses in the cytoplasm (white arrow).

### Mature syncytial cells from the neutrophil, monocyte, and macrophage

**Fig. 4:**
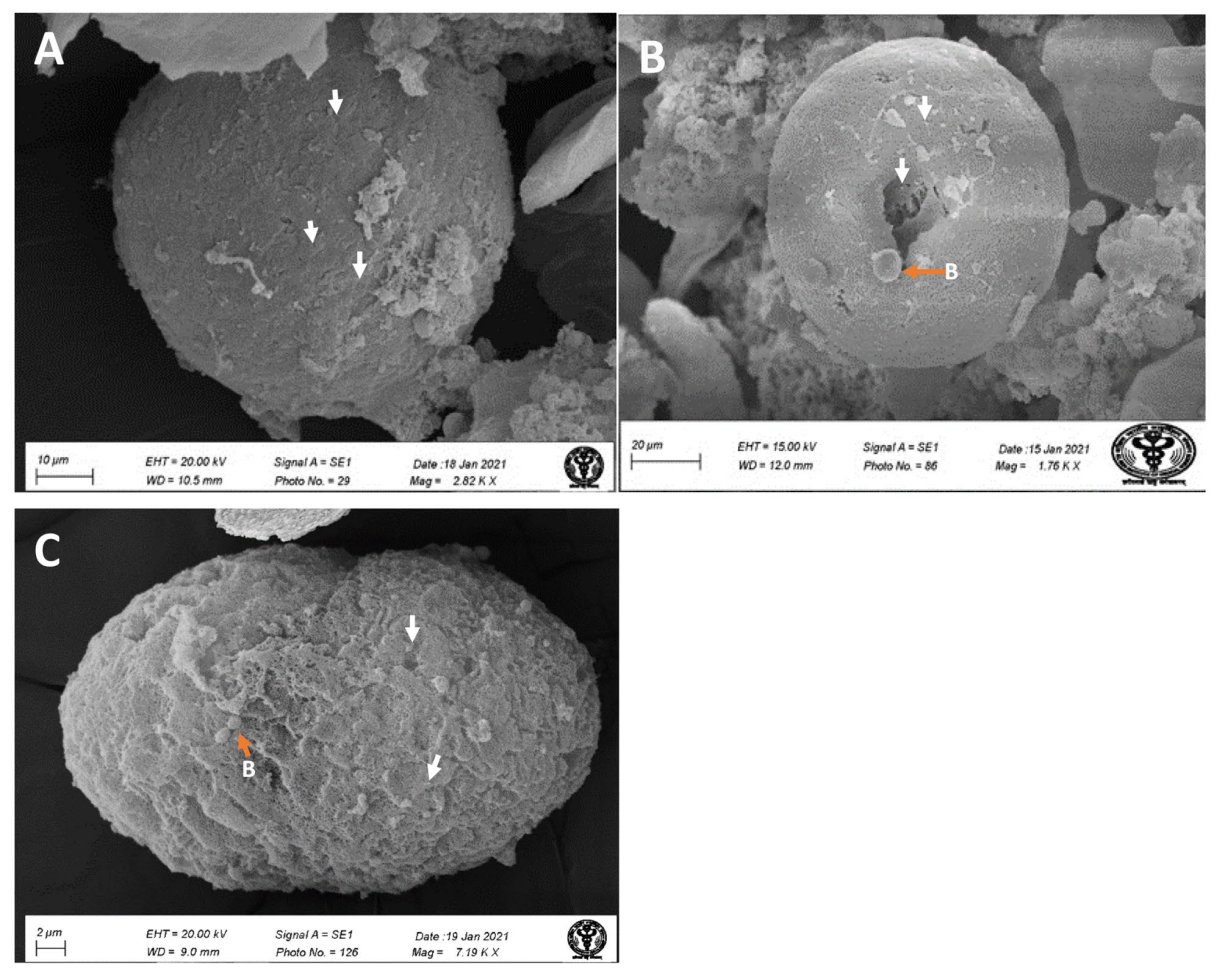
Mature syncytial cells under SEM imaging. Syncytial cells were generated by the fusion of multiple cells of (**A**) neutrophil origin (confirmed by surface projection), (**B**) monocytes (identified by smooth surface), and (**C**) macrophage origin (cytoplasmic projection). ARDS-like COVID-19 patients Mature syncytial cells were observed in severe ARDS-like patients but not in the mild or moderate stage of the disease. White arrow, SARS-CoV-2 virus; B, bacteria (orange arrow).

Matured syncytial cells were not observed in the BALF of mildly and moderately infected patients. However, under SEM imaging, large-size (up to 100 μm) syncytial cells with oval morphology were observed in the severe ARDS-like patients. The ultrastructural surface morphology indicates neutrophils (Fig. 4A), monocytic (Fig. 4B), and macrophage origin (Fig. 4C). The presence of pseudopodia in the plasma membrane and highly folded cell membrane confirms the neutrophilic origin (Fig. 4A). Other matured syncytial cells of monocytic origin were identified by their smooth surface, and the presence of many bacterial-like structures, an indicative of phagocytic cells (Fig. 4B). The surface morphology such as cytoplasmic projections, and wavy structure resemble with the macrophage (Fig. 4C). Such kind of matured syncytia was not observed under TEM.

## Discussion

COVID-19 disease, caused by the severe acute respiratory syndrome coronavirus 2 (SARS-CoV- 2) infection, has become a major threat to global public health [28, 29]. In many patients, the symptoms remain mild (only upper respiratory tract infections with mild fever and without shortness of breath or hypoxia, SpO2 > 95%) and resolve automatically within four to seven days by symptom-based medication. Some patients who developed primary ARDS-like conditions need hospitalization with moderate severity (respiratory rate ≥ 24/min, breathlessness, and SpO2 level 90% to ≤ 93% on room air). A maximum number of patients showed moderate disease-like symptoms between 8 and 15 days after the first symptoms. However, 2–5% of COVID-19 patients developed severe ARDS-like conditions with frequent breathing problems, low SpO2 (< 90%), and high CT scores (> 16) [30, 31]. A higher number of patients were reported after the 17th day of the disease. Syncytia formation is associated with the severity of COVID-19. However, it is possible that syncytia formation may also be related to the duration of the infection, as well as other factors such as the patient’s immune response and viral load. It is still not fully understood how syncytia formation contributes to the pathogenesis of COVID-19. Thus, we have taken both parameters as disease severity and the day of sample collection after the infection to equalize the patient conditions.

Histological imaging of lung autopsy samples (death due to COVID-19) showed multinucleate giant syncytial cells [12, 15, 16, 20]. However, it is difficult to explore the initiation of syncytia formation and its correlation with the disease stage from these autopsy samples. This is because light microscopy-based histological techniques are unsuitable for identifying the respiratory and hemopoietic cells involved in syncytia formation. Our electron microscopy-based ultrastructural study of BALF samples from mildly infected COVID-19 patients did not find syncytia (Additional file 1. Figure S1). This indicates that SARS-CoV-2 viruses might not initiate membrane fusion at the initial stage of the disease. This may be because the virus tends to multiply, tropism, and infects the maximum number of cells to propagate, including the oral and respiratory epithelium, in the initial phase of the disease [32, 33]. At this stage, the virus does not need special facilitation for its replication, dissemination, immune evasion, and inflammatory responses because the host immunological response was not yet activated efficiently. However, in the moderate stage of the disease, a negative pressure was created on the replication and dissemination of the SARS-CoV-2 virus by activating humoral and cellular immunological responses [34]. Macrophage activation and granulocyte infiltration in lung tissue (neutropenia) force the virus to activate its safeguard mechanism to facilitate its replication and dissemination through the fusion of the plasma membrane of respiratory and hemopoietic cells [20, 35, 36]. This could be a reason for plasma membrane fusion with identical and heterotypic cells at the moderate stage of the disease (Figs. 1, 2 and 3). Entirely fused binucleate type II pneumocyte syncytial cells were observed in this disease stage (Fig. 1). However, in a similar stage of the disease, incomplete plasma membrane fusion (initiation of membrane fusion) was observed in hemopoietic origin cells such as monocytes and neutrophils (Figs. 2 and 3). This indicates that type II pneumocytes were the first choice of the virus to induce syncytial cell formation, which may help in the replication and protection of the virus from the immune response [21]. This could be the reason for high fluorescence (indicative of high infection and multiplication) in type II pneumocyte-origin syncytial cells (Fig. 1). A recent study also supported this, which provided critical insights and the consequences of multinucleated giant cell formation after SARS-CoV-2 infections [16, 20]. Further, the formation of homologous syncytia was also reported *in vitro* Vero cells (ACE2+) expressing SARS-CoV-2 spike protein [21].

It is hypothesized that syncytia may also contribute to viral dissemination and immune evasion by protecting the virus from immune cells and from neutralizing antibodies [1, 12]. In our study, the initiation of identical (in neutrophils) and heterotypic cell fusion (with neutrophils and monocytes) in the moderate stage of the disease indicates the hyperactivation of viral fusogen proteins to initiate the syncytia formation in these hematopoietic cells (Figs. 2 and 3). It was reported that the viral spike (S) protein worked as a fusogen on the surface of an infected cell which interacts with receptors on neighboring cells to form syncytial cells [37]. Besides its ability to drive the fusion of viral and cellular membranes, the S protein can further drive the fusion of neighboring cells, which results in the formation of multinucleated giant cells [12, 14, 15]. This finding was also supported by a recent study in which SARS-CoV-2-induced syncytia targeted the lymphocytes for internalization and cell-in-cell mediated elimination, potentially contributing to lymphopenia and pathogenesis in COVID-19 patients [20].

The finding of mature and very large size syncytial cells (20–100 μm) of neutrophils and monocytes origin at very late and severe stages (ARDS-like) of the diseases indicated the role of syncytial cells in the viral dissemination and immune evasion. These giant syncytial cells may help in the multiplication and protection from the body’s immune response, which may be the reason for the alveolar macrophage activation and, ultimately, the cytokine storm [20]. Mature syncytial cells were not identified in our TEM imaging though it was reported in the autopsy samples. This could be attributed to the release of fewer such kinds of cells from the infected lung tissue to the BALF of the patients.

## Conclusions

Syncytia formation is an important step to protect and help in the multiplication of the SARS-CoV-2 virus after efficient immunological activation of patients. The current study showed that syncytia formation was first induced in type II pneumocytes by homotypic fusion and later with haematopoetic cells (monocyte and neutrophils) by heterotypic fusion in the moderate stage (9-16 days) of the disease. The syncytia matured in the late phase of the disease and formed large giant cells of 20 to 100 μm. This detailed ultrastructural study on single and different cell origins of syncytia may explore the degree to which syncytial cells contribute to the pathology and provide insight into the disease biology of COVID-19.

## Electronic supplementary material

Below is the link to the electronic supplementary material.

Supplementary Material 1

Supplementary Material 2

## Data Availability

Biosamples numbers and patients detail are listed in Additional file 1: Supplementary Table 1.
