# Supplementary material for: Ultrastructural study confirms the formation of single and heterotypic syncytial cells in bronchoalveolar fluids of COVID-19 patients
Source: Virol J. 2023 May 19;20:97. doi: 10.1186/s12985-023-02062-7 (PMC10198030; doi:10.1186/s12985-023-02062-7)
Supplement: Supplementary file 1 — Supplementary Material 1 [file 12985_2023_2062_MOESM1_ESM.docx]

**Enlarged view of each image given in the main text figure (Specially for Reviewer):**


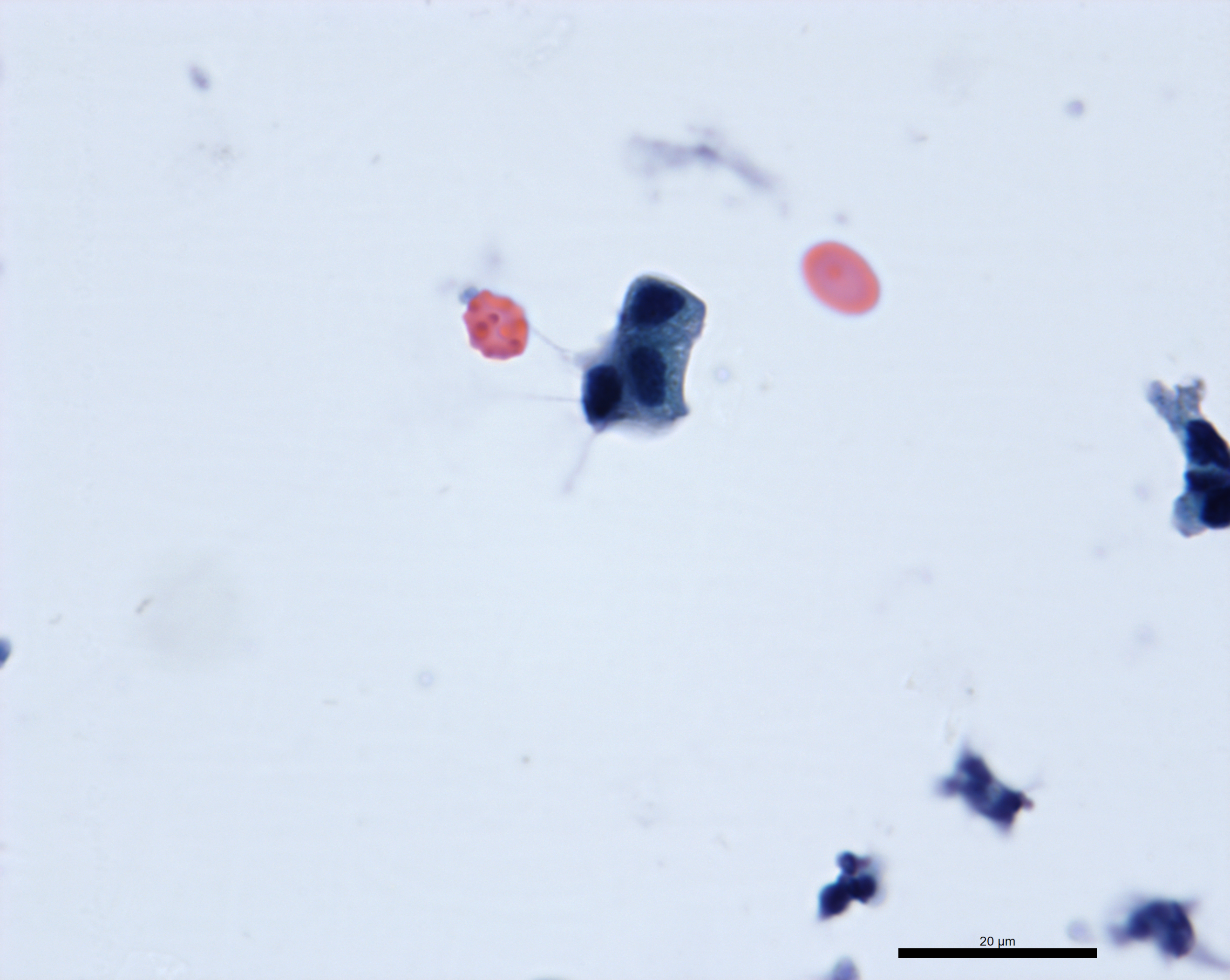


5um

**N**

**A**

**Fig.1**


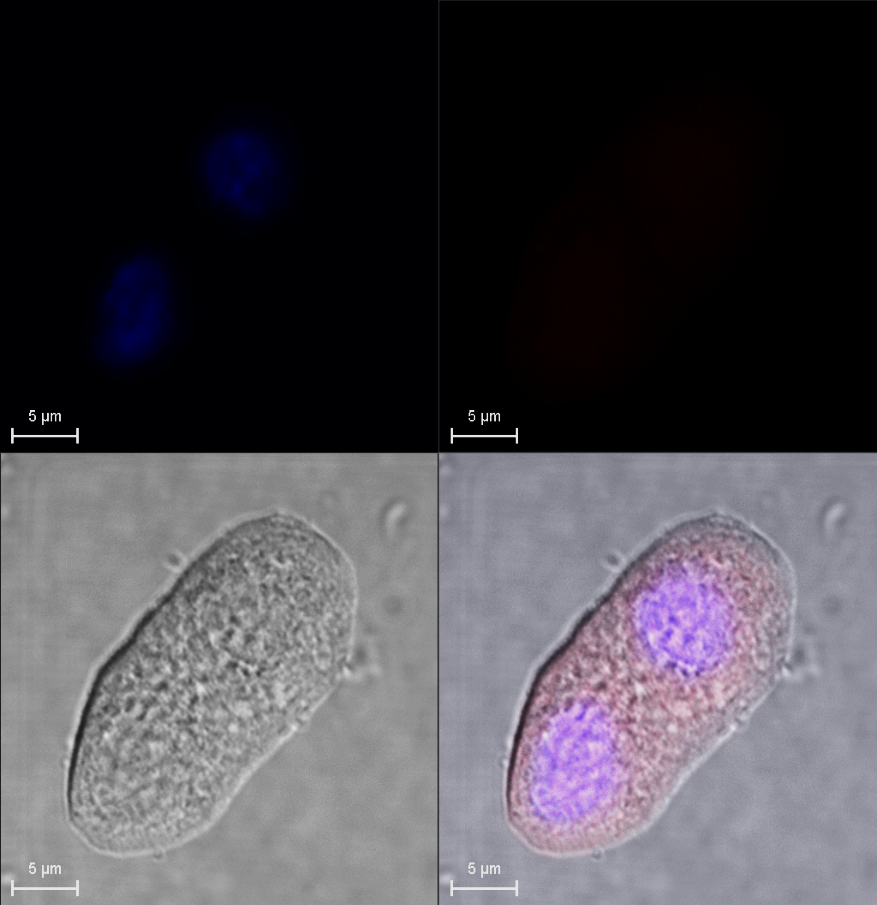


**N**

**B**

**microvilli**





**microvilli**

**C**

**Fig.2**


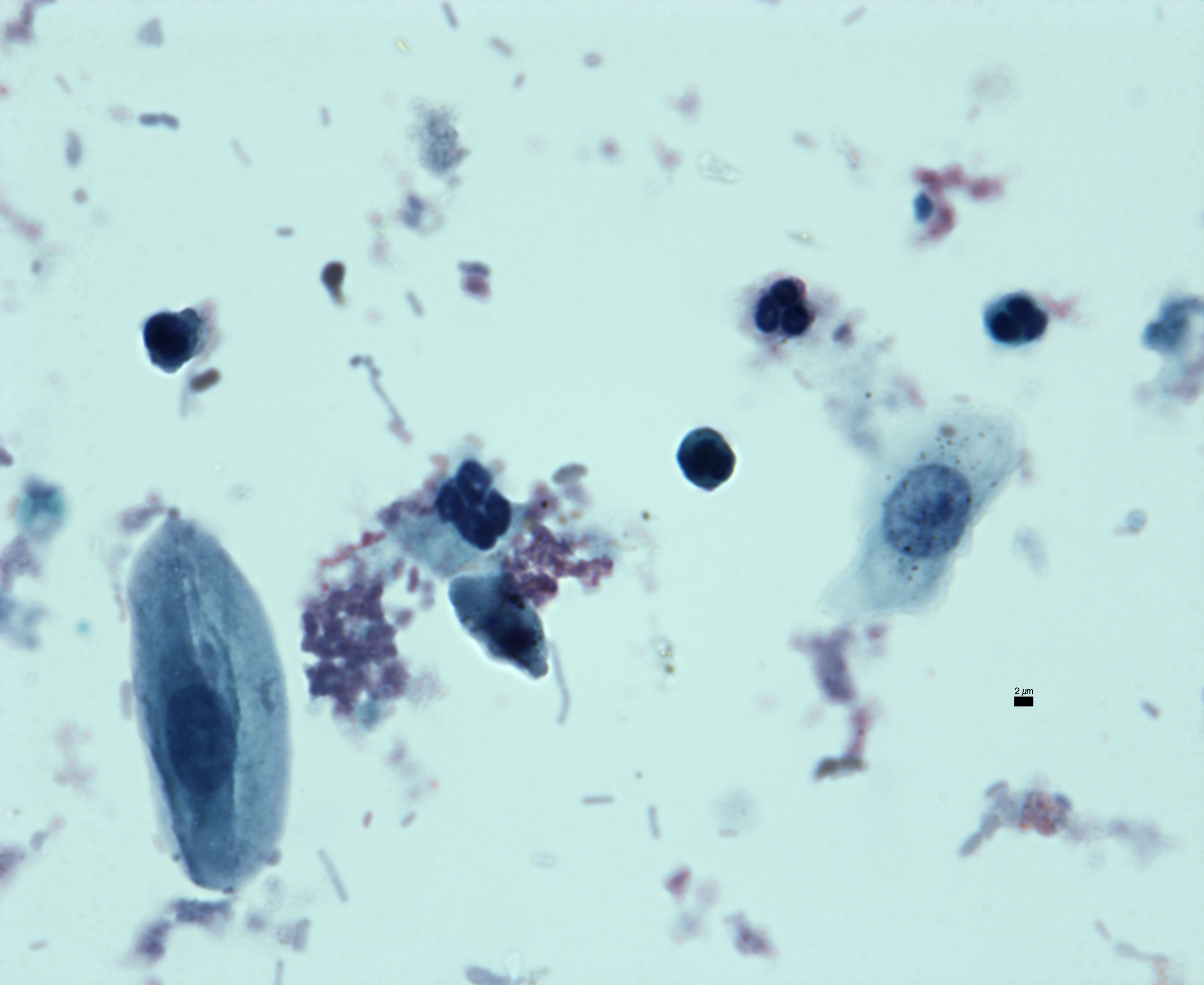


5um

**N**

**A**


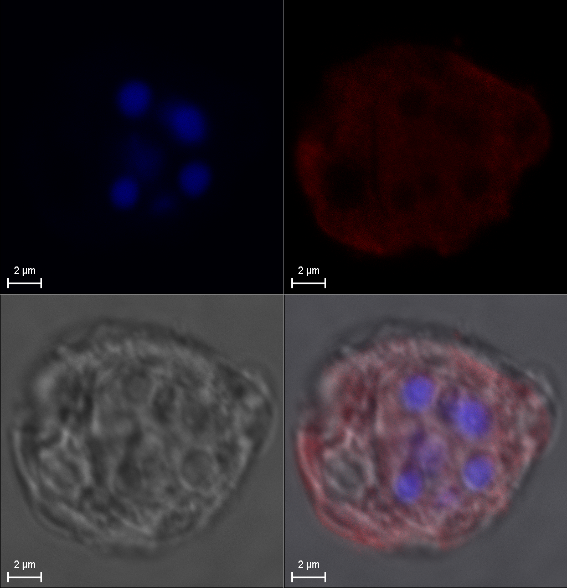


**N**

**B**





**C**


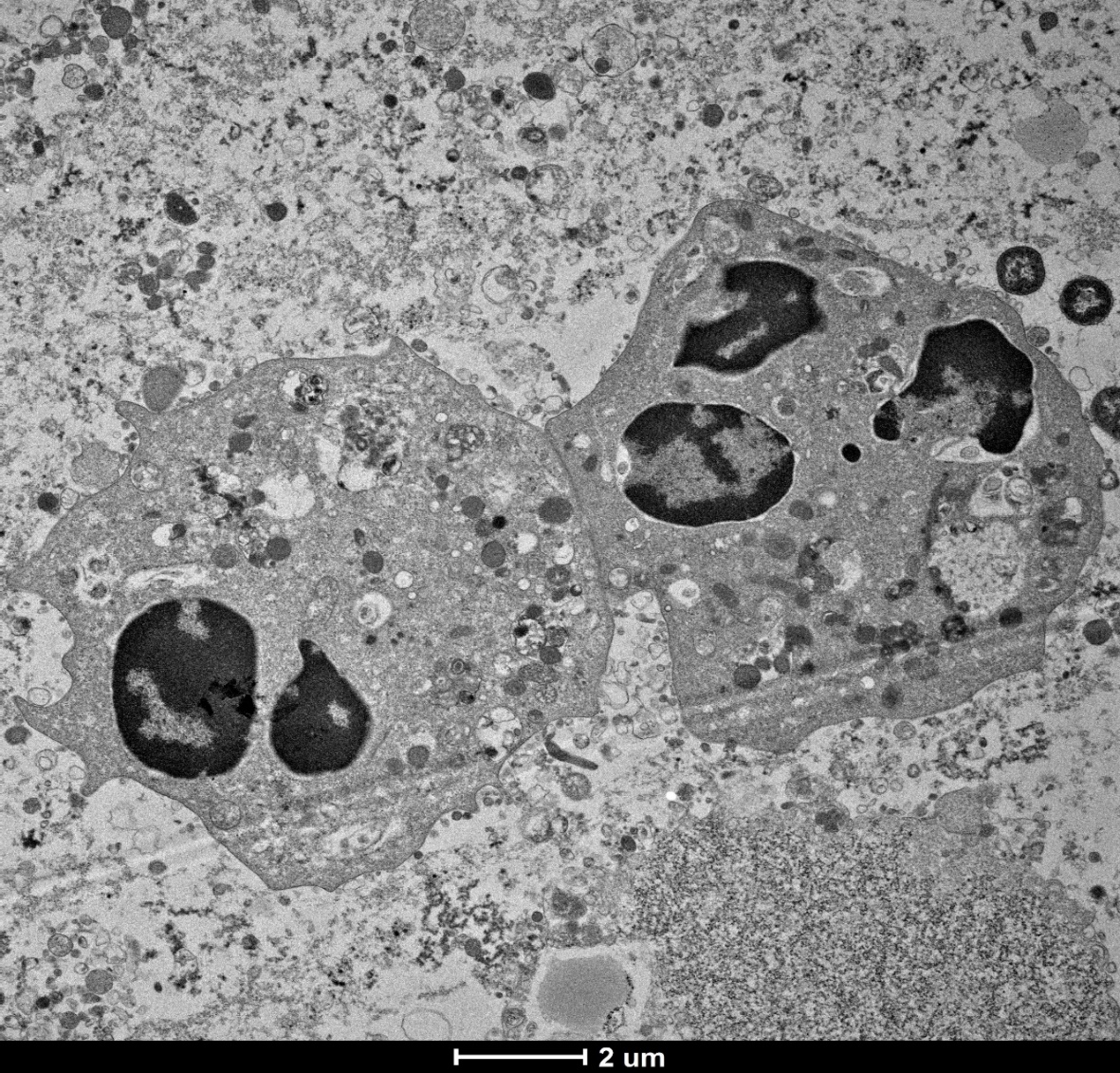


**N**

**N**

**M**

**RB**

**P**

**RB**

**M**

**RB**

**Pse**

**D**

**B**


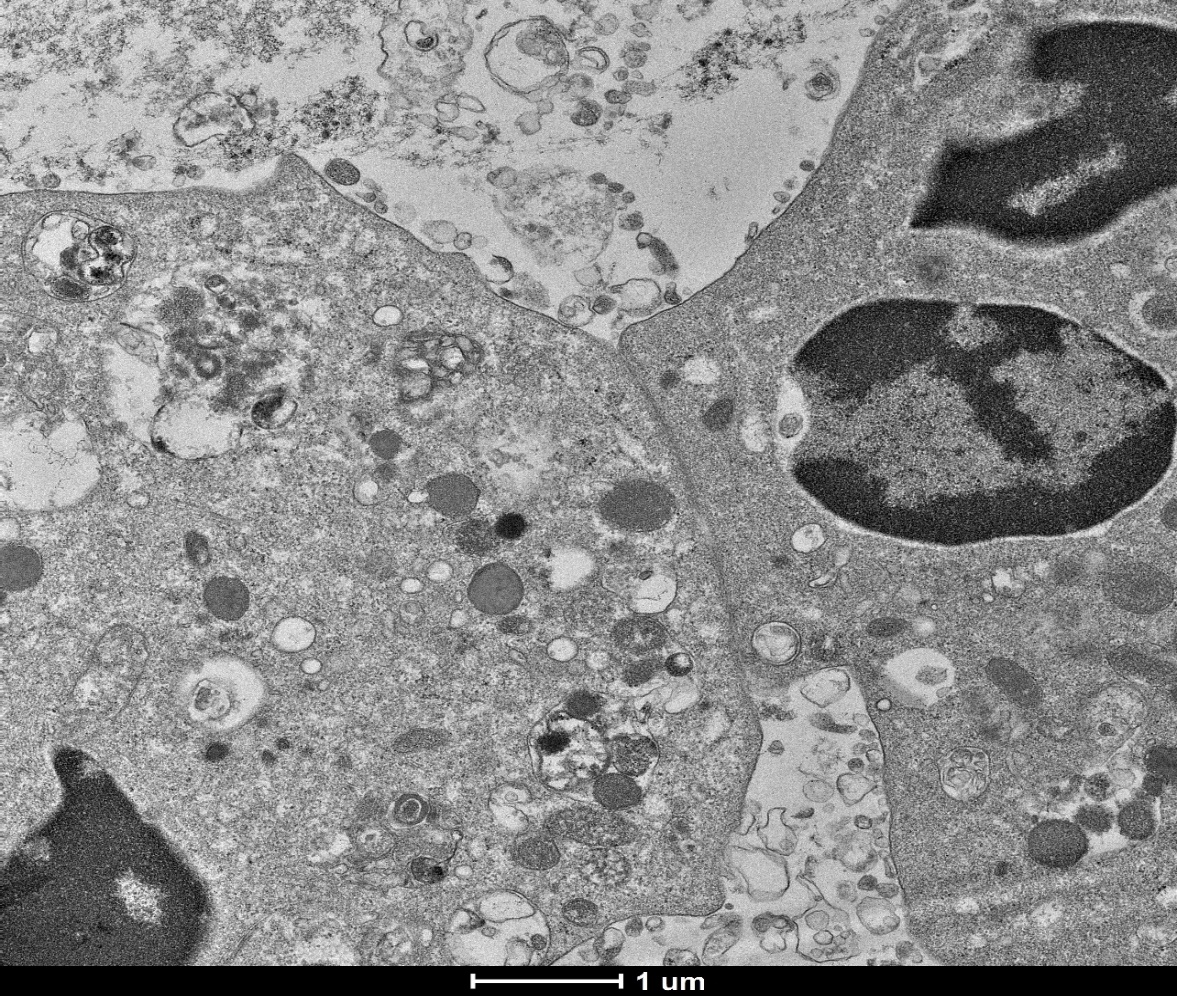


**RB**

**M**

**RB**

**RB**

**RB**

**N**

**N**

**M**

**E**


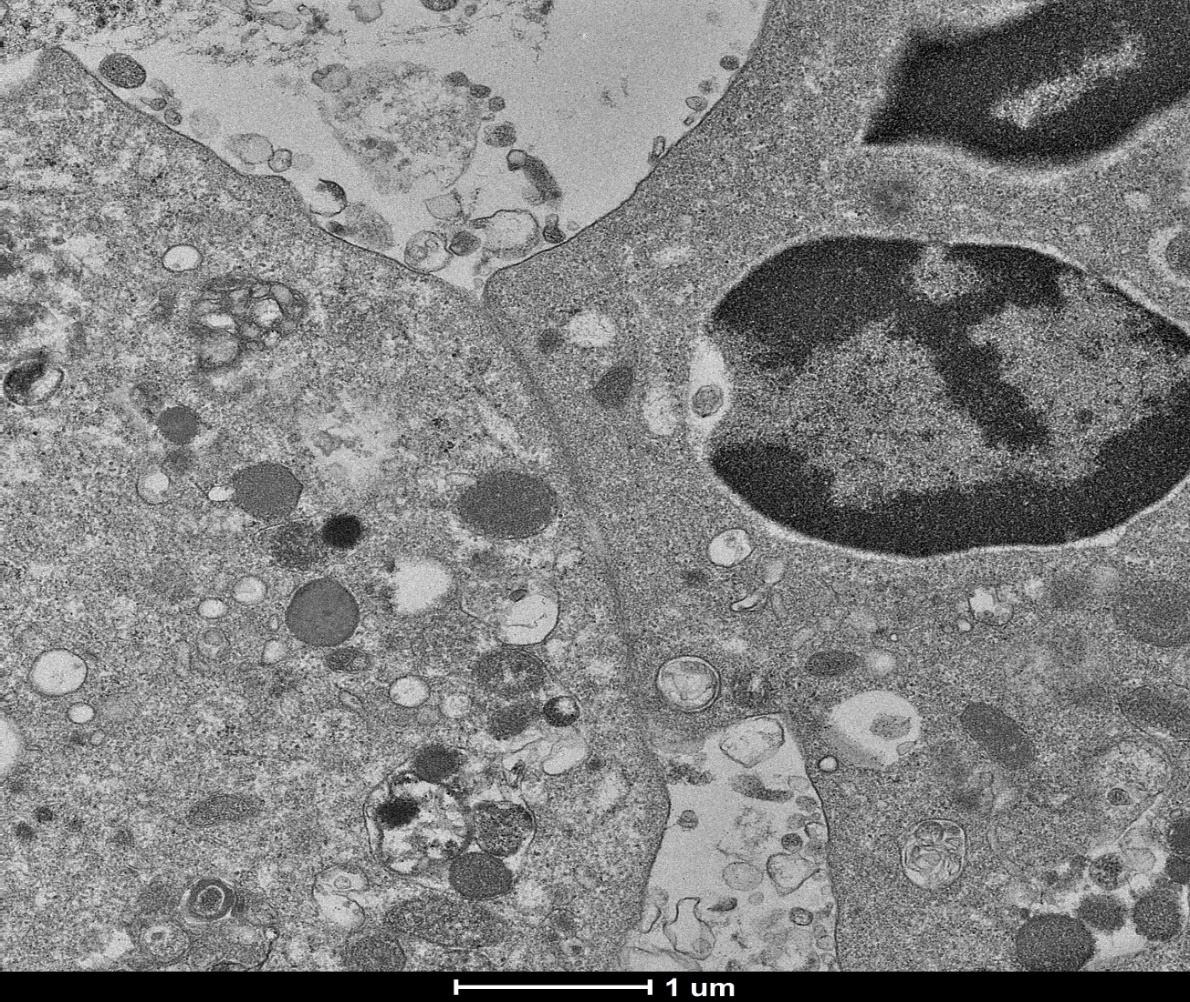


**RB**

**RB**

**RB**

**N**

**F**

**M**

**Fig.3**


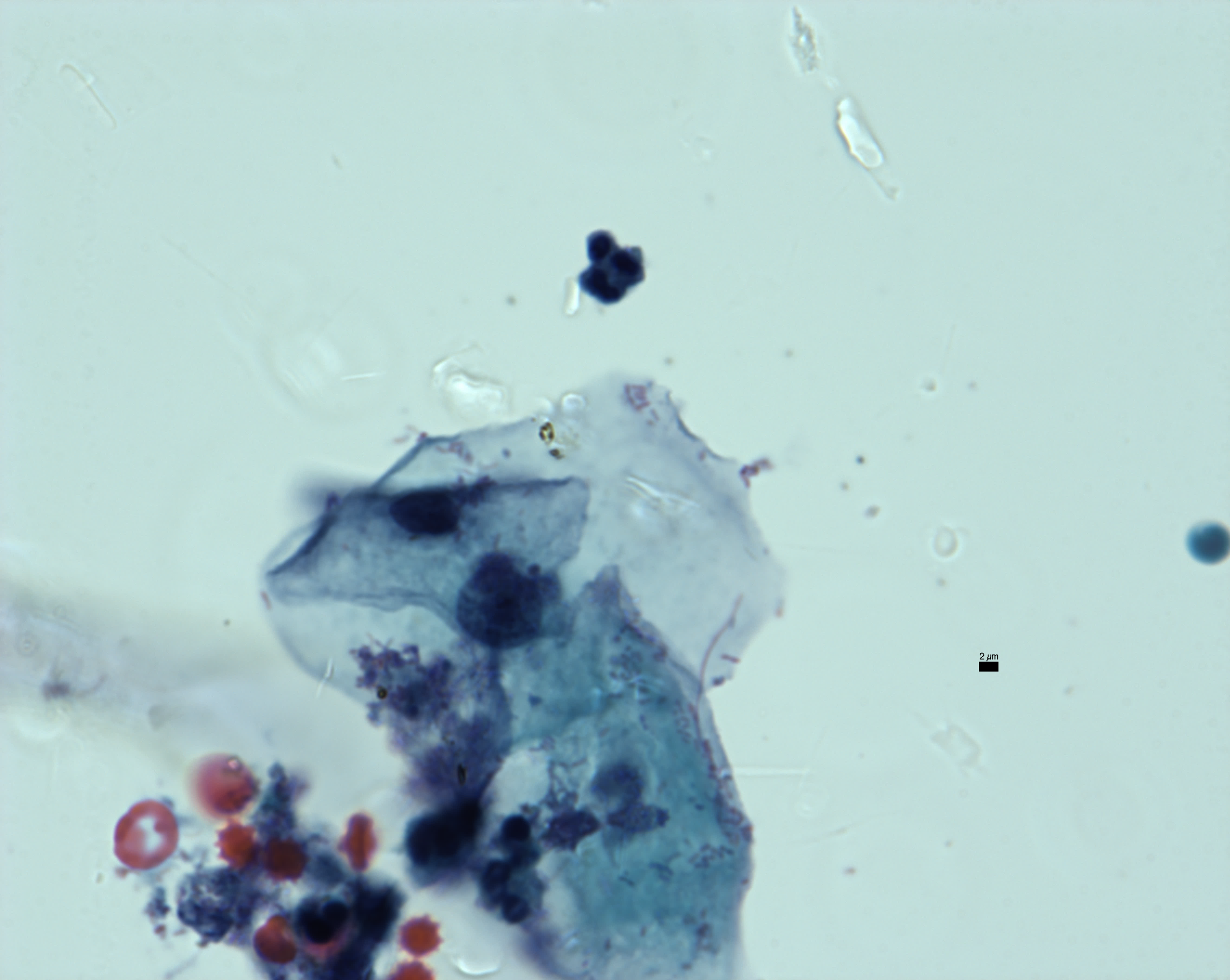


5um

**N**

**A**


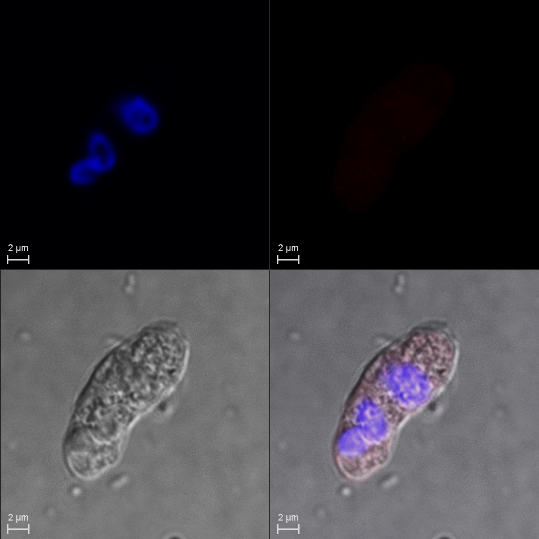


**N**

**B**

**Mo**

**Nt**


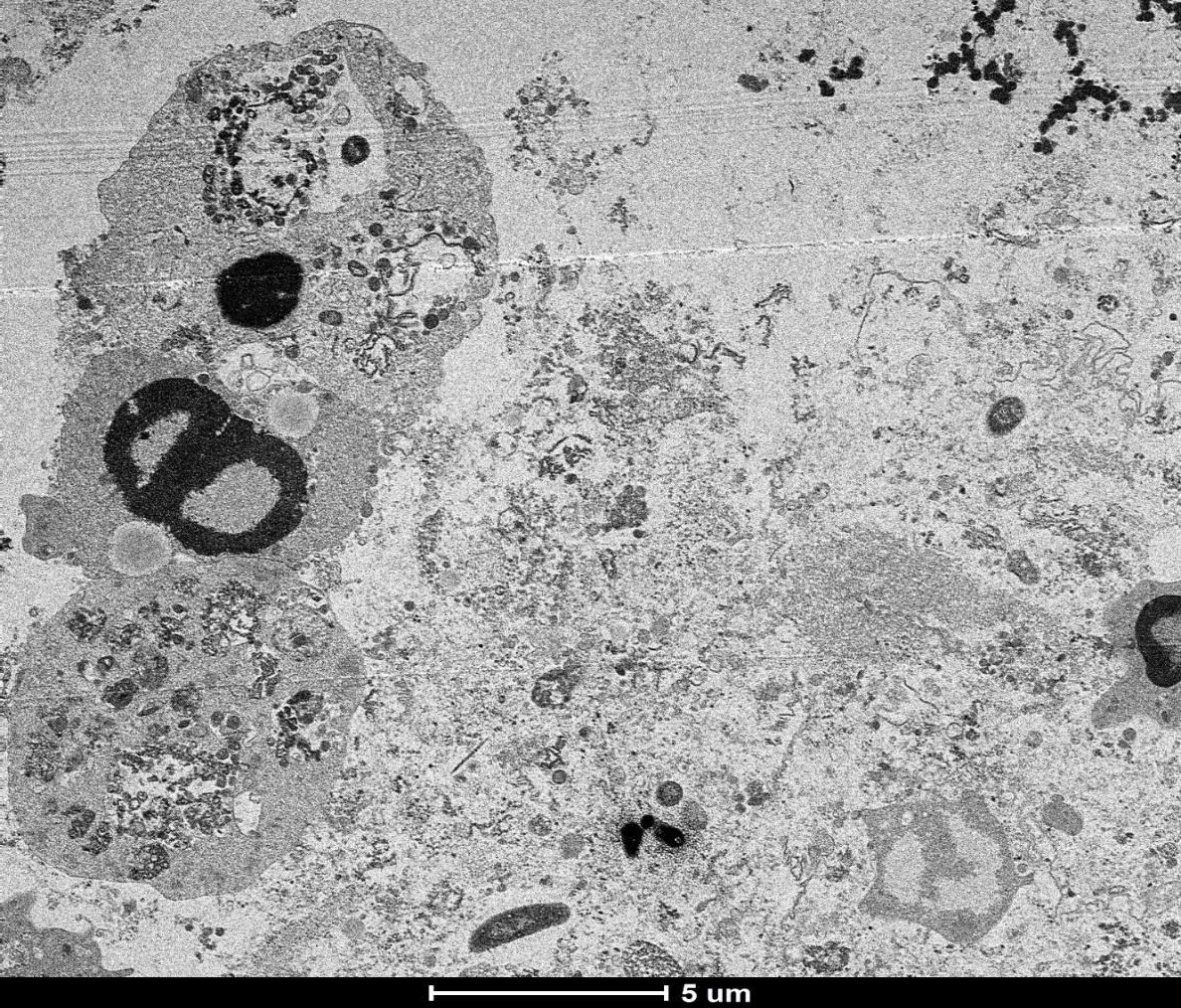


**N**

**N**

**Neutrophil**

**Neutrophil**

**Monocyte**

**B**

**C**


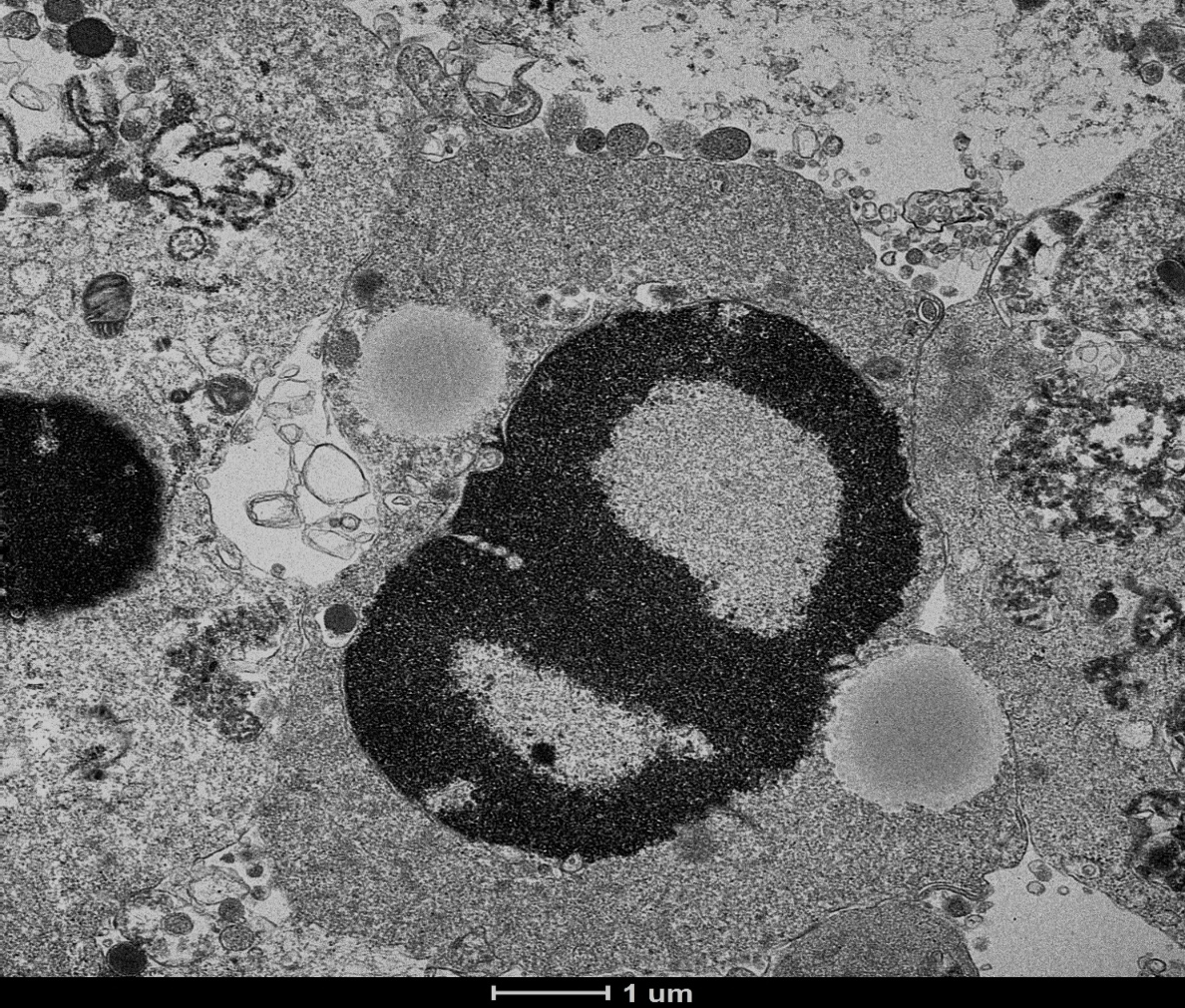


**RB**

**RB**

**L**

**L**

**M**

**D**

**N**


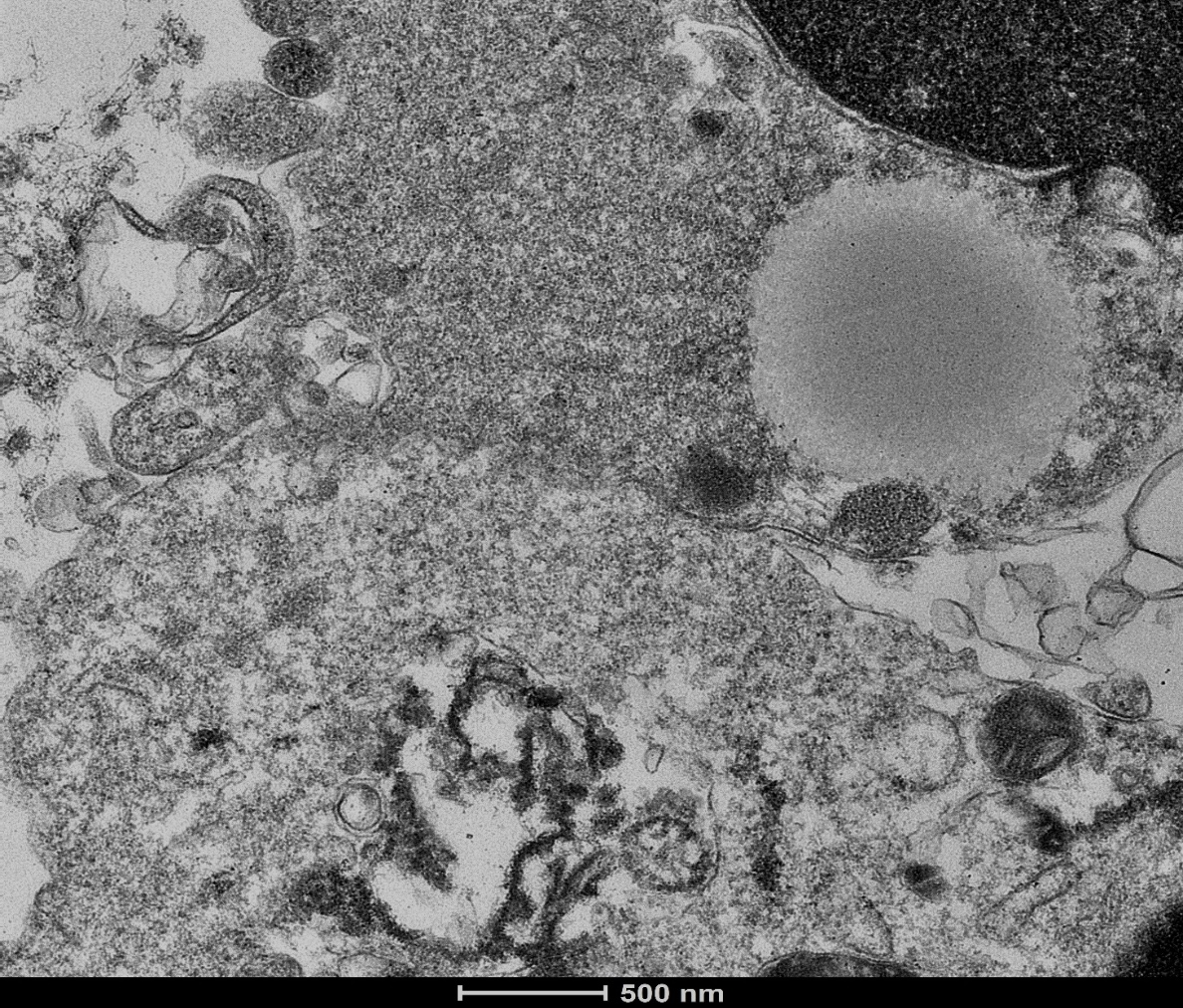


**L**

**M**

**RB**

**E**

**RB**

**L**


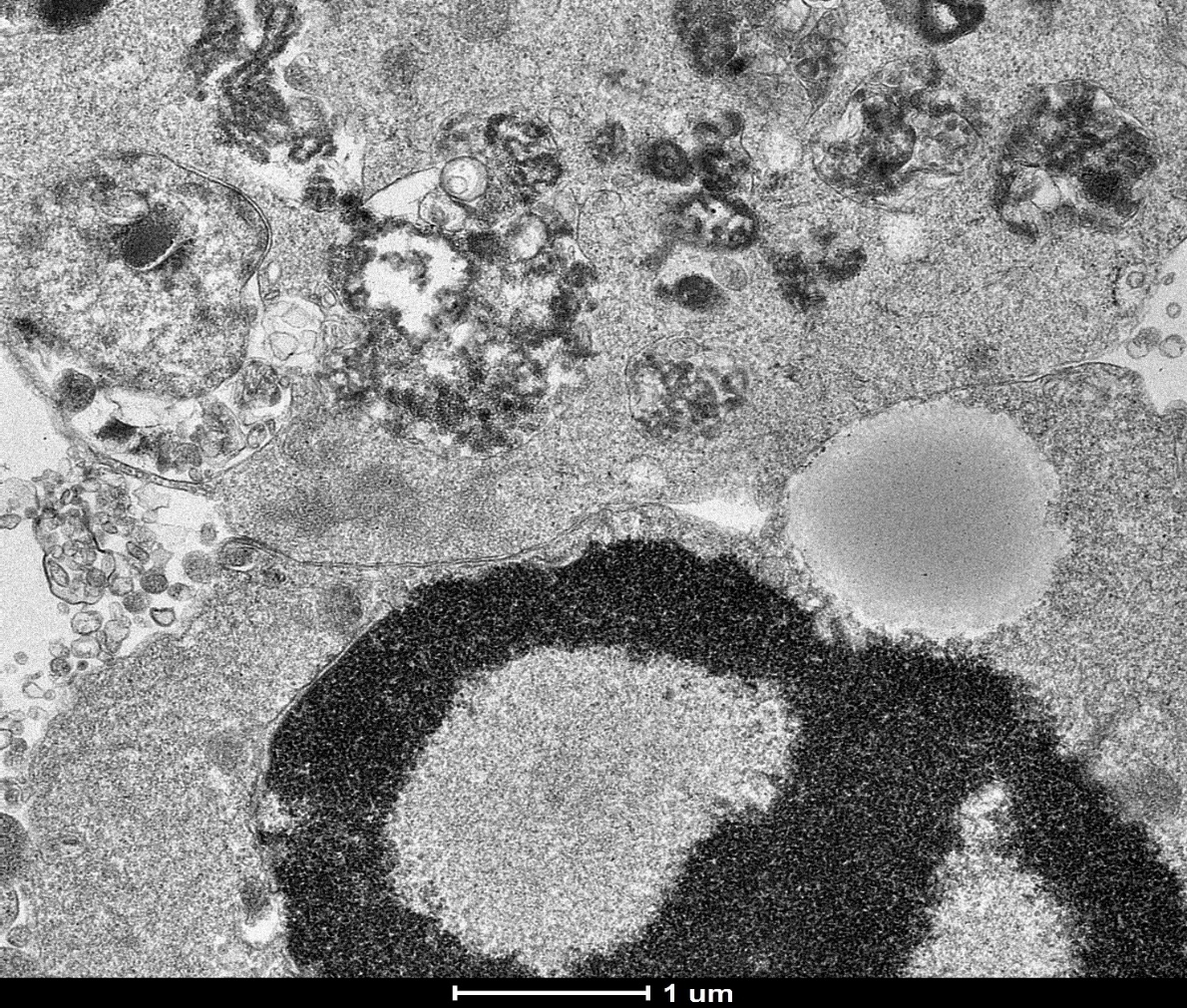


**L**

**RB**

**N**

**F**

**L**

**RB**

**Fig.4**


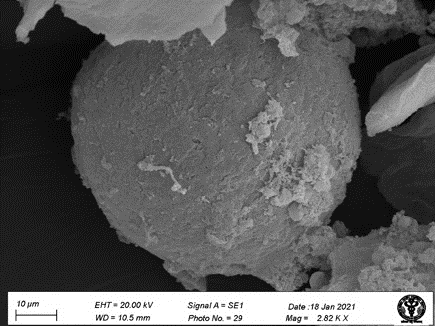


**A**


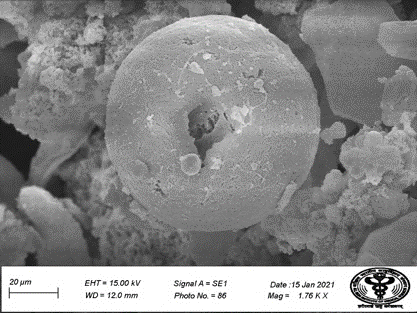


**B**

**B**


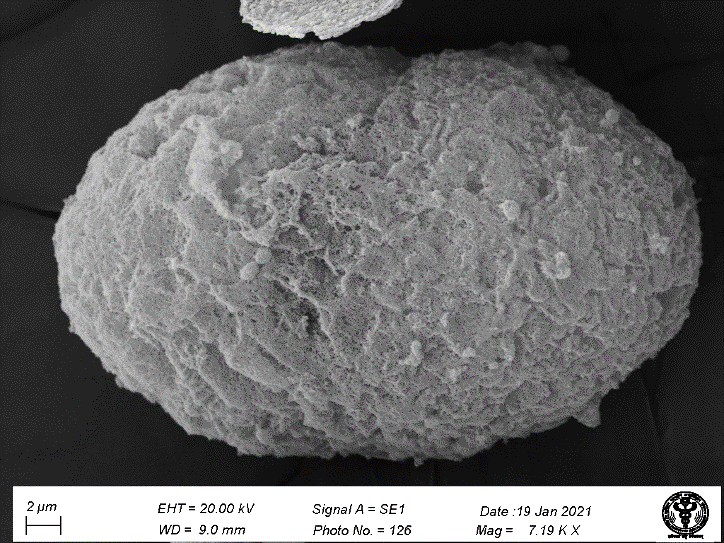


**C**

**B**

**N**

**N**

**N**
