# Supplementary material for: Ultrastructural study confirms the formation of single and heterotypic syncytial cells in bronchoalveolar fluids of COVID-19 patients
Source: Virol J. 2023 May 19;20:97. doi: 10.1186/s12985-023-02062-7 (PMC10198030; doi:10.1186/s12985-023-02062-7)
Supplement: Supplementary file 2 — Supplementary Material 2 [file 12985_2023_2062_MOESM2_ESM.docx]

**Supplementary Figure S1:**


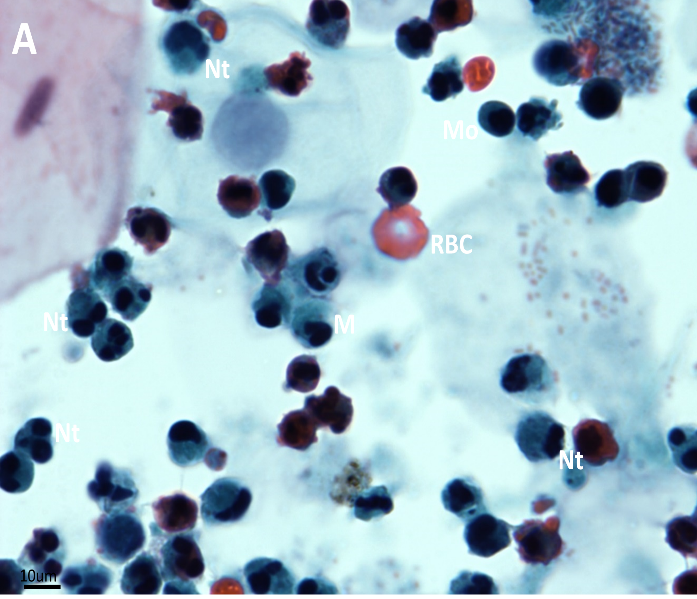

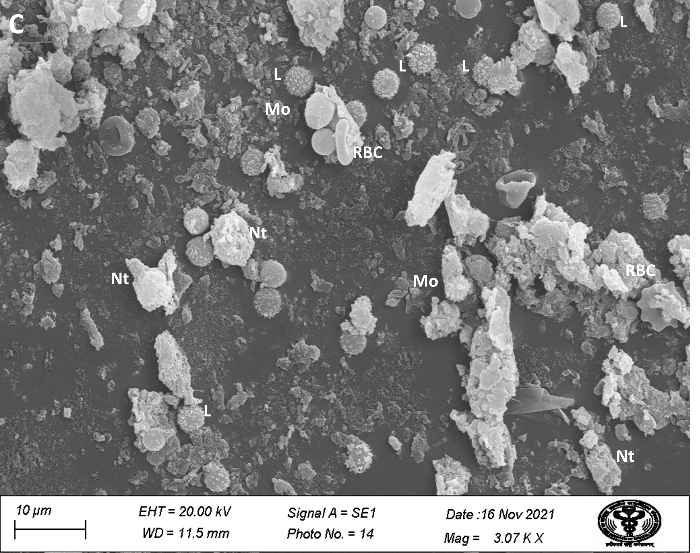

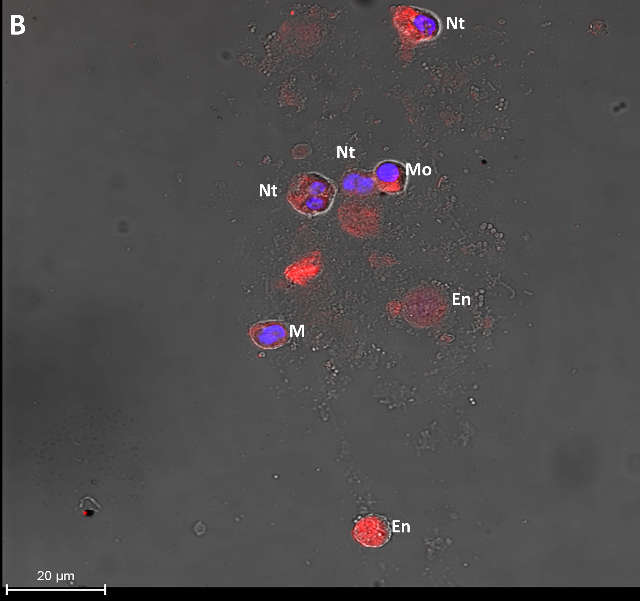

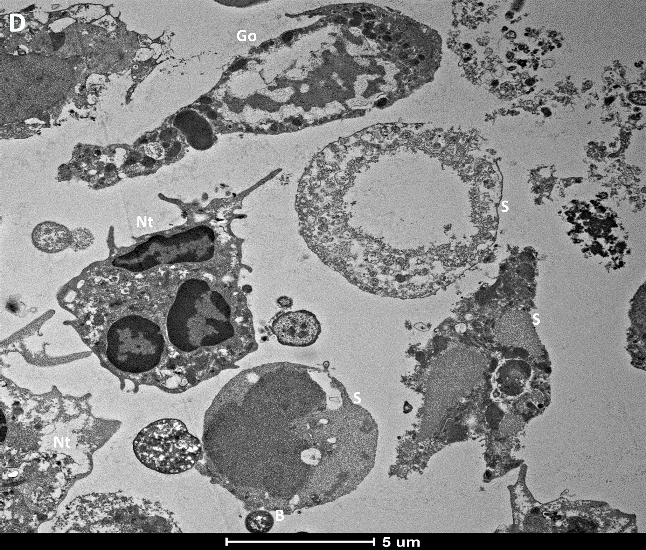


**Supplementary Fig. 1. PAP, IF, SEM, and TEM imaging of mildly infected patients with COVID-19.** These images did not show any syncytia. M, macrophage; Mo, monocyte; Nt, neutrophil; L, lymphocyte; Go, Goblet cell; S, squamosal cells; En, enucleated granulocytes; RBC, red blood cell.

**Supplementary Table S1: Detail of the recruited patients, their classification, and clinicopathological data**

| **S. No** | **Age** | **Gender** | **Covid -positive Date** | **Date of Sample Collection** | **D-dimer (<0.5 µg/mL)** | **IL-6 (5-15 pg/mL)** |
| --- | --- | --- | --- | --- | --- | --- |
| **Mild Infection patients ( between 2 to 8 days after infection)** | | | | | | |
| 1 | 60 | F | 2020.11.05 | 2020.11.06 | 6.1 | NA |
| 2 | 75 | M | 2020.12.30 | 2021.01.03 | 8.39 | 118.55, 214 |
| 3 | 62 | F | 2021.02.06 | 2021.02.12 | NA | 16.30, 98.59 |
| 4 | 62 | F | 2021.02.06 | 2021.02.14 | NA | 16.30, 98.59 |
| 5 | 33 | M | 2020.10.30 | 2020.11.06 | 2.1 | 29.11, 10.49, 22.23 |
| 6 | 21 | M | 2021.01.02 | 2021.01.09 | 12, 6.62 | 16.20, 86.47 |
| 7 | 35 | M | 2021.02.02 | 2021.02.07 | NA | 84.38 |
| 8 | 11 | M | 2021.02.10 | 2021.02.12 | NA | 132.98,30.56, 115.28,182.34 |
| **Moderate Infection patients ( between 9 to 16 days after infection)** | | | | | | |
| 1 | 60 | F | 2020.09.23 | 2020.10.09 | NA | NA |
| 2 | 69 | M | 2020.12.28 | 2021.01.09 | 14.45 | 1166.77,746.38 |
| 3 | 48 | M | 2020.12.14 | 2020.12.29 | 2.38, 3.85 | 371, 1620, 378.43 |
| 4 | 23 | M | 2021.01.14 | 2021.01.23 | 2.24 | 27.56, 77,81, 9.90 |
| 5 | 41 | M | 2021.01.29 | 2021.02.07 | NA | 33.90, |
| 6 | 50 | F | 2020.09.27 | 2020.10.11 | NA | 19.86, 57.38 |
| 7 | 45 | F | 2020.12.20 | 2020.12.29 | 2.1, 2.90 | 77.66, 7.36, 8.43 |
| 8 | 42 | F | 2021.01.23 | 2021.02.07 | NA | 54.89, 49.98, 4.40 |
| **Severe Infection patients ( between 17 days and above after infection)** | | | | | | |
| 1 | 65 | M | 2020.12.20 | 2021.01.09 | 1.58 | 38.10, 2.90, 3.42 |
| 2 | 67 | M | 2021.01.20 | 2021.02.07 | NA | 9.58, 26.71, 1066.97 |
| 3 | 67 | M | 2021.01.20 | 2021.02.12 | NA | 76.56 |
| 4 | 67 | M | 2021.01.20 | 2021.02.14 | 5.23 | 26.71, 9.58 |
| 5 | 50 | M | 2020.09.20 | 2020.10.22 | 3.35, 2.88, 3.96 | 95.76, 129, 374.26 |
| 6 | 38 | M | 2021.01..06 | 2021.02.14 | NA | NA |
| 7 | 50 | M | 2020.12.31 | 2021.01.23 | NA | 42.36, 127, 106 |
| 8 | 43 | F | 2020.12.28 | 2021.01.23 | 19.22 | 284.96 |
